# Novel albumin-binding photothermal agent ICG-IBA-RGD for targeted fluorescent imaging and photothermal therapy of cancer†

**DOI:** 10.1039/d0ra09653a

**Published:** 2021-02-11

**Authors:** Cheng Yu, Enhua Xiao, Pengfei Xu, Jingjing Lin, Linan Hu, Jie Zhang, Shengzhen Dai, Zhuyuan Ding, Yuyang Xiao, Zhu Chen

**Affiliations:** Departments of Radiology, The Second Xiangya Hospital, Central South University Changsha Hunan 410011 P. R. China chenzhu415@csu.edu.cn; Department of Psychiatry, The Second Xiangya Hospital, Central South University, National Clinical Research Center for Mental Disorders, Hunan Medical Center for Mental Health Changsha Hunan 410011 P. R. China; Institute of Clinical Pharmacy & Pharmacology, Jining First People's Hospital, Jining Medical University Jining 272000 P. R. China

## Abstract

In this work, we present a novel photothermal agent ICG-IBA-RGD based on albumin-binding strategy for enhanced tumor targeting imaging and photothermal therapy. *In vitro* and *in vivo* experiments demonstrated that ICG-IBA-RGD exhibits excellent photothermal conversion capability and high tumor ablation efficiency.

## Abstract 2

In this work, we present a novel photothermal agent ICG-IBA-RGD based on albumin-binding strategy for enhanced tumor targeting imaging and photothermal therapy.

## Graphical abstract

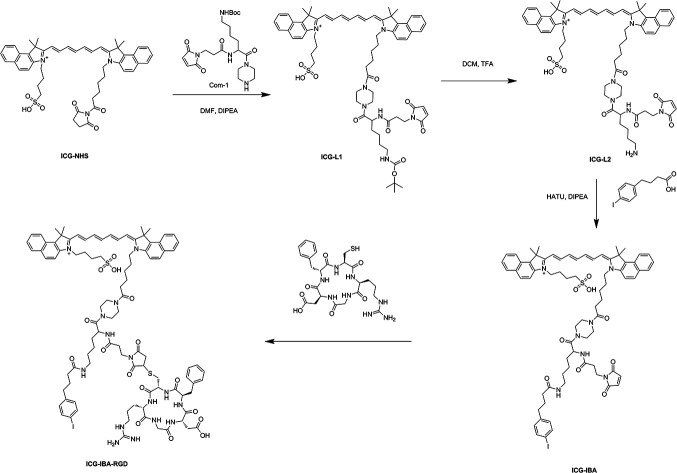

## Introduction

Nowadays, cancer has been becoming a leading cause of human death with the rapid population growth and aging around the world.^1^ Conventional clinical treatments for cancer, including surgical intervention, chemotherapy and radiotherapy, still have their deficiencies. Surgery treatment cannot remove all tumor cells, and sometimes even leads to the spread of tumor cells. Due to the lack of tumor specificity, both radiotherapy and chemotherapy will induce severe local or systemic side effects during tumor therapy.^2^ Besides, tumor tissue or cells will also become resistant to chemotherapeutic drugs and radiation during chemotherapy irradiation processes. Based on the limitations of the above therapeutic methods, photothermal therapy (PTT), which is a novel non-invasive cancer treatment strategy, has attracted extensive attention because of its high efficiency, easy operation, negligible side effects and good bioavailability.^3,4^ The core of PTT is photothermal agents (PTAs) that can convert near-infrared (NIR) light into cytotoxic heat for killing tumor cells.^5^ Importantly, PTT can ignore the effects of cellular resistance because it induces cell death *via* physical mechanisms such as protein denaturation and membrane rupture.^6^ Additionally, it can realize high precision since thermal effect is produced only when NIR light and PTAs are combined.

Extensive efforts have been made to develop numerous types of inorganic and organic PTAs. Inorganic nanomaterials including gold nanoparticles,^7,8^ sulfide nanoparticles,^9,10^ and carbon-based materials^11,12^ exhibit favorable absorbance feature, excellent photothermal conversion efficiency and good photo-stability. However, obstacle of metabolism and potential long-term toxicity may restrict their future clinical translation.^13^ It has been shown that gold nanoparticles can retain in the mononuclear phagocyte system for over 6 months and induce adverse effects including DNA damage, oxidative stress and inflammation.^14–16^ Organic small molecule-based PTAs with good biocompatibility and biodegradability offer distinct advantages with regard to potential clinical application,^17^ which have attracted increasing attention as potential alternatives to nanomaterials in the field of PTT. Indocyanine green (ICG) is a NIR dye for FL and PA imaging approved by the U.S. Food and Drug Administration (FDA). Furthermore, ICG shows its potential as photothermal agent as its satisfactory NIR optical absorption and photothermal conversion capabilities. Therefore, ICG has been employed as a kind of theranostic platform in many studies.^18–20^

However, the instability in aqueous medium and nonspecific biodistribution *in vivo* of ICG hamper its clinical use as photothermal agent.^21^ To overcome these limitations, ICG loaded nanoparticles which can improve the stability of ICG against light and heat and enhance specific tumor targeting ability should be developed. Human serum albumin (HSA) is the most abundant protein in plasma with multiple ligand binding sites, cellular receptor engagement, and a long circulatory half-life (19 days) because of interaction with the recycling neonatal Fc receptor.^22–25^ The utilization of these properties promotes albumin to be an attractive candidate for half-life extension and targeted intracellular delivery of drugs attached by covalent conjugation, genetic fusions, association or ligand-mediated association.^26^ As the long circulating protein in the blood, albumin has been used to extend blood half-life and reduce renal clearance of both imaging probes and therapeutic drugs.^27^ In addition to the prolonged half-life, albumin has also been found to specifically target tumor regions due to its enhanced permeability and retention (EPR) effect, abnormal nutritional needs, albumin receptor binding and SPARC-inducing effect,^28–30^ which is a unique advantage as the tumor-targeted drug delivery carrier.^31,32^ Dumelin *et al.*^33^ reported a class of albumin binders with stable non-covalent binding to HSA, of which 4-(*p*-iodophenyl)butyric acid (4-IBA) was one of the promising structures and aroused great attention in the field of molecular imaging and cancer radioligand therapies. The category of 4-IBA derivatives was first identified by Neri's group. These binders exhibited stable non-covalent binding with albumin and dissociation constants (*K*_d_) in the low micromolar range. In addition, since the binding of 4-IBA to albumin is reversible, the albumin–drug complex can be used as a drug reservoir to enhance the drug biodistribution and bioavailability.^26^ It has been proved that 4-IBA-modified radio pharmaceuticals have extremely high affinity to HSA, and improve the biological half-life and tissue distribution *in vivo*.^34–36^ However, the application of 4-IBA as the albumin binder to improve the half-life and therapeutic efficacy of PTAs has rarely been reported.

**Scheme 1:**
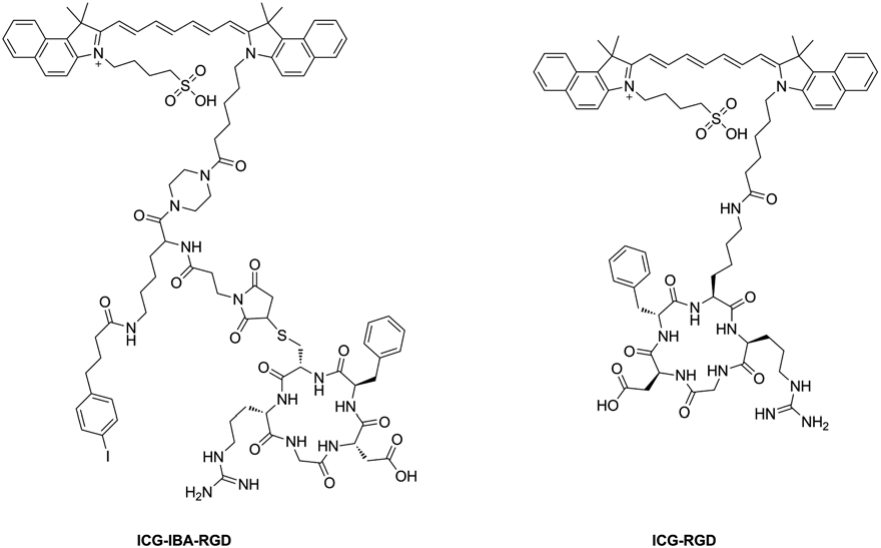
The structure of probe ICG-IBA-RGD and ICG-RGD.

In this work, we report a novel photothermal agent termed as ICG-IBA-RGD for efficacy NIR imaging and image-guided therapy (Scheme 1). ICG-IBA-RGD is comprised of three parts: (1) ICG for NIR imaging and photothermal therapy; (2) c-RGD for targeting α_v_β_3_-integrin overexpressed on tumor angiogenic endothelium; (3) 4-IBA for albumin binding. ICG-IBA-RGD exhibited good biocompatibility, excellent photothermal conversion capability upon single 808 nm laser irradiation. *In vitro* and *in vivo* experiments demonstrated that ICG-IBA-RGD could efficiently ablate tumor cells and completely eliminate tumor through photothermal effect when irradiated with an 808 nm laser. Notably, the tumor did not recur during the experiment. In summary, this work presents a biocompatible multifunctional albumin-binding photothermal agent ICG-IBA-RGD with great potential for image-guided photothermal therapy.

## Results and discussion

### Preparation and characterization of ICG-IBA-RGD

**Scheme 2:**
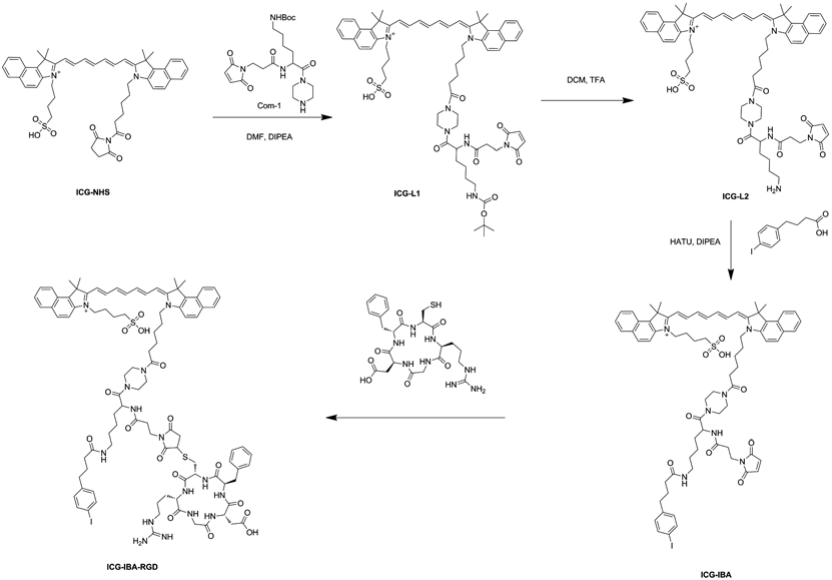
The synthesize route of probe ICG-IBA-RGD.

The synthetic route of ICG-IBA-RGD was shown in Scheme 2. ICG-L1 can be easily achieved by ICG-NHS and compound 1. Followed deprotection of Boc group of the intermediate ICG-L1 by neat TFA, 4-IBA was introduced to ICG-L2. Then, the free thiol group of RGD was covalently attached to the maleimide motif of ICG-IBA to give ICG-IBA-RGD.

### Photothermal performance of ICG-IBA-RGD

**Fig. 1:**
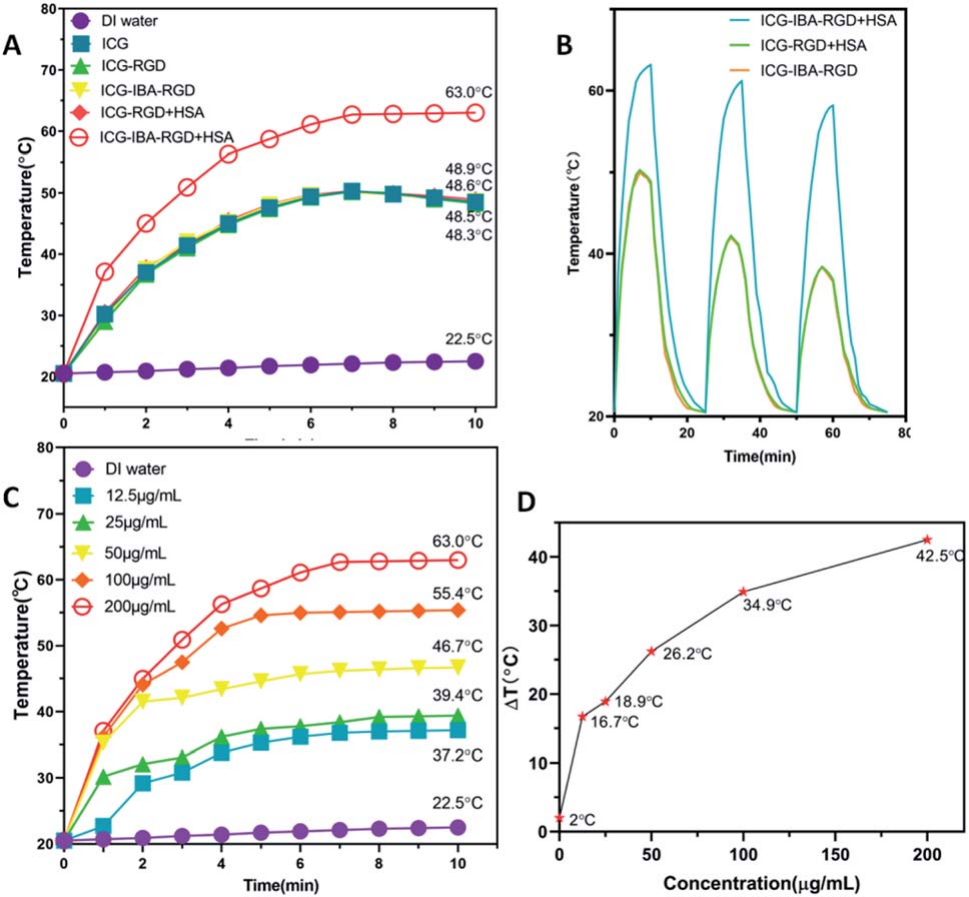
(A) Photothermal increasing temperature curves of ICG-IBA-RGD + HSA, ICG-RGD + HSA, ICG-IBA-RGD, ICG-RGD, ICG and deionized water with the irradiation time. (B) The photothermal stabilities of ICG-IBA-RGD + HSA and ICG-RGD + HSA solutions after three cycles of 808 nm laser (1 W cm^−2^) on/off. (C) Temperature of ICG-IBA-RGD + HSA at different concentrations upon laser irradiation for 10 min. (D) Temperature variation (Δ*T*) of ICG-IBA-RGD + HSA at different concentrations upon laser irradiation for 10 min.

In order to study the photothermal performance of ICG-IBA-RGD, different solutions, including ICG-IBA-RGD + HSA, ICG-RGD + HSA, ICG-IBA-RGD, ICG-RGD, ICG and deionized water, were irradiated for 10 min by an 808 nm NIR laser at a power density of 1.0 W cm^−2^. As shown in Fig. 1A, ICG-IBA-RGD + HSA showed the highest temperature increment within 10 min, while the temperature increases for the corresponding ICG-RGD + HSA, ICG-IBA-RGD, ICG-RGD and ICG were almost same. As shown in Fig. 1B, the temperature increments for ICG-IBA-RGD + HSA did not change much with the cycle times, whereas the temperature increments for ICG-RGD + HSA and ICG-IBA-RGD decreased significantly. This proved that the binding of IBA and HSA improved the photothermal stability of ICG-IBA-RGD. Additionally, gradient concentrations of ICG-IBA-RGD + HSA suspensions (0, 12.5, 25, 50, 100, 200 μg mL^−1^) were exposed to 808 nm laser (1.0 W cm^−2^) irradiation for 10 min. As shown in Fig. 1C, the concentration and irradiation time-dependent temperature of ICG-IBA-RGD + HSA suspension increased significantly under laser irradiation. Specifically, the temperature of 200 μg mL^−1^ suspension of ICG-IBA-RGD + HSA remarkably raised from approximately 20.5 °C to 63 °C after 10 min of irradiation, which is high enough to kill tumor cells *via* hyperthermia. Fig. 1D showed the temperature change (Δ*T*) of ICG-IBA-RGD + HSA suspensions at various concentrations.

### *In vitro* cytotoxicity and cell-killing effect of ICG-IBA-RGD

**Fig. 2:**
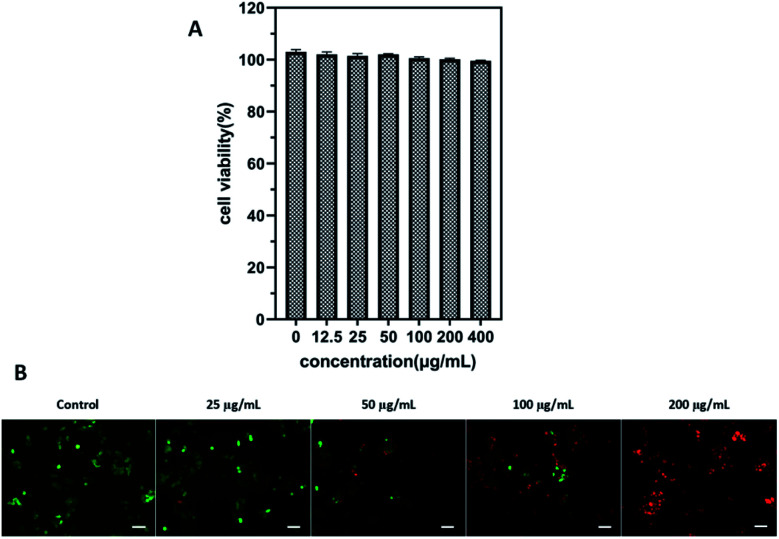
(A) Cell viability of 4T1 cells after incubation with ICG-IBA-RGD + HSA at different concentrations for 24 h. (B) Fluorescence images of 4T1 cells upon NIR irradiation with different concentrations of ICG-IBA-RGD. Green: FDA, live cells; red: PI, dead cells. Scale bar: 100 μm.

Cell viability experiment was performed in 4T1 cells through the CCK-8 assay to evaluate the cytotoxic effect of ICG-IBA-RGD. After incubation with ICG-IBA-RGD + HSA for 24 h, no significant toxicity (cell viability was above 90%) was observed on 4T1 cells even at the highest concentration of 400 μg mL^−1^ (Fig. 2A). The results indicate that the ICG-IBA-RGD possess excellent biocompatibility and very low biotoxicity *in vitro*.

To further verify cell-killing effects of ICG-IBA-RGD in conjunction with NIR laser irradiation, 4T1 cells treated with different conditions were stained with a fluorescein diacetate (FDA, green fluorescence for live cells) and propidium iodide (PI, red fluorescence for dead cells) to visualize live and dead cells. As shown in Fig. 2B, 4T1 cells treated with either 808 nm laser irradiation alone exhibited bright green fluorescence in the entire area, indicating that the cell viability was not compromised. By contrast, 4T1 cells treated with ICG-IBA-RGD + HSA plus laser irradiation showed red fluorescence. The number of dead cells increased with the increase of the ICG-IBA-RGD concentration. At the concentration of 100 μg mL^−1^, the 4T1 cells were obviously dead, and at 200 μg mL^−1^, there were no living cells in the field of view. The results suggest that the high photothermal conversion efficiency of ICG-IBA-RGD could effectively kill tumor cells *in vitro*.

### Tumor-targeted fluorescence imaging and biodistribution *in vivo*

**Fig. 3:**
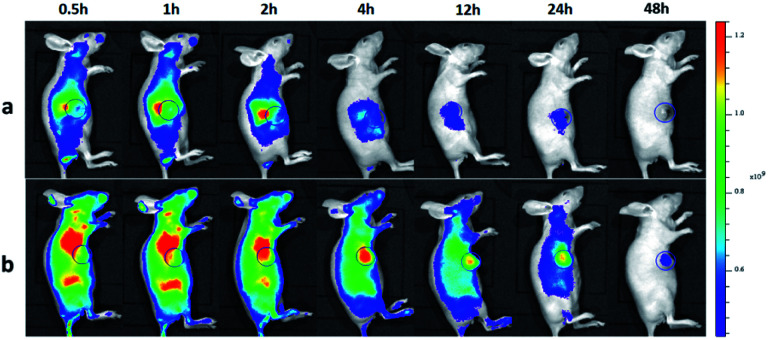
*In vivo* imaging and biodistribution analysis of mice bearing 4T1 tumors after tail vein injection ICG-RGD (a) and ICG-IBA-RGD (b) (the tumors are circled with a blue circle line).

To investigate the tumor-targeting ability of ICG-IBA-RGD*in vivo*, intravenous injections of ICG-IBA-RGD and ICG-RGD through the tail vein to the mice bearing 4T1 tumors were performed, and then the intensity of the fluorescence emitted by the live animal was monitored by the *in vivo* imaging system. As shown in Fig. 3, the color bar from blue to red was used to imply the increase of fluorescent intensity, and the tumor was marked by a circle area. In the ICG-IBA-RGD treated group, after intravenous injection, majority of the drugs were retained in the blood circulation due to the stable interaction of ICG-IBA-RGD with serum albumin. After 0.5 h, ICG-IBA-RGDs were mainly distributed in the liver and kidney, indicating that the liver and kidney are the main organs for drug metabolism and elimination. After 1 h, ICG-IBA-RGDs began to accumulate in the tumor area. After 2 h, the accumulation of ICG-IBA-RGDs in tumor area was gradually increased, while drugs in liver and kidney were gradually removed. After 4 h, the accumulation of ICG-IBA-RGDs in the tumor area reached the peak, while most of the drugs in the liver and kidney were removed. In the following 4 to 48 h, the ICG-IBA-RGDs in the blood circulation gradually decreased, while the accumulation of ICG-IBA-RGDs in tumor area still maintained a high level. The enhanced accumulation can be explained by both specific retention of albumin in the tumor tissue as well as the EPR effect. By contrast, time-dependent fluorescence images of mice after the injection of ICG-RGD showed that the relatively weak signal was detected in the tumor area due to the fast blood clearance and the relatively poor tumor accumulation. These results indicated that ICG-IBA-RGD can target the tumor site more effectively. Fig. S5† indicates *ex vivo* FL images of major organs and tumors harvested from mice at the time of 48 h. As expected, for the ICG-RGD group, few fluorescence was detected in the tumor, while higher fluorescence intensity was detected in the liver, which is the major metabolic organ of ICG-RGD. In the ICG-IBA-RGD group, a greater amount of fluorescence was detected in the tumor. Moreover, the weak fluorescence intensity of major organs, such as heart, liver, spleen, lung and kidney, further validated the tumor oriented accumulation of ICG-IBA-RGD. The fluorescence in liver was mainly concentrated in the gallbladder area. The above results indicated that ICG-IBA-RGD can effectively reveal its distribution and confirm tumor location to realize efficient PTT.

### *In vivo* tumor therapeutic effect

**Fig. 4:**
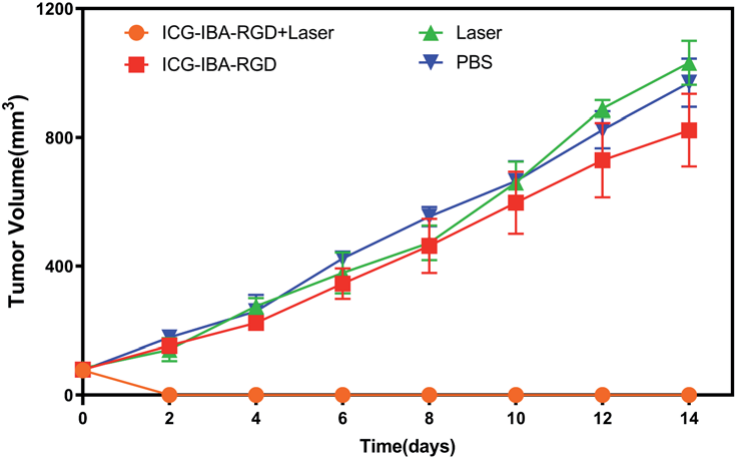
Tumor growth curves of tumor-bearing mice after various treatments (*n* = 3).

**Fig. 5:**
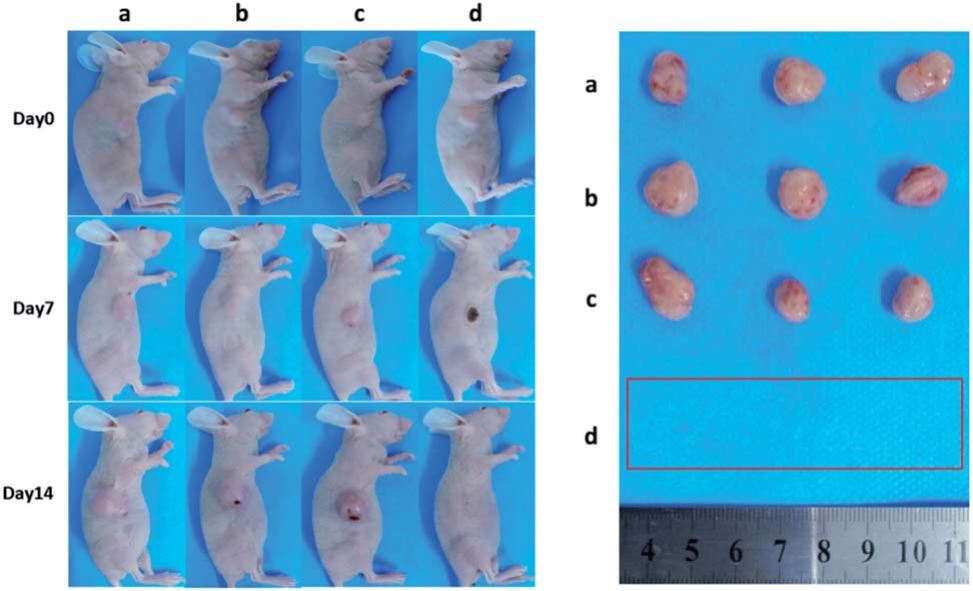
Photographs of representative BALB/c mice and extracted tumors after being treated with (a) PBS, (b) laser, (c) ICG-IBA-RGD, and (d) ICG-IBA-RGD + laser, respectively.

Encouraged by above results, the PTT experiments *in vivo* are then performed on 4T1 tumor model. As mentioned before, the mice were divided randomly into 4 groups: (1) PBS only (2) laser only (3) ICG-IBA-RGD (1 mg mL^−1^) (4) ICG-IBA-RGD (1 mg mL^−1^) + laser. Throughout a 14 day treatment period, the size of the tumors was measured every 2 days and the growth curve of tumors was drawn. As shown in Fig. 4, tumors treated with PBS only, laser only and ICG-IBA-RGD kept natural growth trend. By comparison, the tumors of mice treated with ICG-IBA-RGD and irradiated with the laser exhibited complete tumor elimination and no tumor recurrence during the experiment. The satisfactory outcome benefited from the powerful PTT effect and outstanding tumor accumulation of ICG-IBA-RGD. Fig. 5 showed the representative photographs of mice and tumors treated with different methods, which were consistent with the results above. In addition, the steady increase in the body weight of all four groups implied no adverse effects from the PTT treatment (Fig. S6†). The biochemistry test results showed that the changes were negligible in liver and kidney function markers, including alanine aminotransferase (ALT), aspartate aminotransferase (AST) and blood urea nitrogen (BUN), demonstrating no injury of the treatments on liver and kidney function (Fig. S7†). Meanwhile, H&E staining also showed no significant acute injury or adverse effects in the main organs (heart, liver, spleen, lung and kidney) for each group (Fig. S8†), which further demonstrated the favorable biocompatibility of ICG-IBA-RGD.

**Fig. 6:**
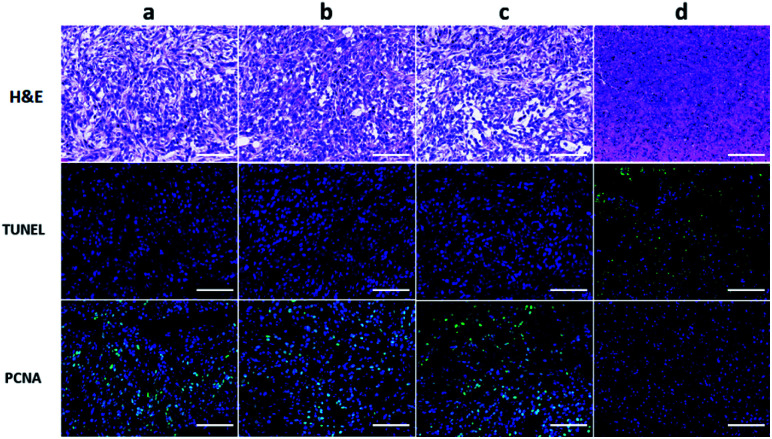
H&E, TUNEL and PCNA staining for tumor section from different treated groups after 14 days post treatment. The green fluorescence indicates the TUNEL or PCNA signal. Scale bar: 100 μm ((a) PBS; (b) laser; (c) ICG-IBA-RGD; (d) ICG-IBA-RGD + laser).

To further evaluate the efficacy of ICG-IBA-RGD on tumor PTT, the histopathological sections of tumor of the mice in different groups were analyzed 14 days after treatment. Histological images of H&E stained tumor sections showed that there was no obvious malignant necrosis in the groups of mice treated with the PBS, laser and ICG-IBA-RGD only, while apparent nucleus dissociation and necrosis were observed in mice treated with ICG-IBA-RGD plus laser. In addition, the TUNEL staining results showed that the apoptotic cells of tumor tissues had significantly increased in ICG-IBA-RGD treated mice with 808 nm laser irradiation, but not in the other three groups. PCNA staining results indicated that the proliferation capacity of tumor tissue was obviously reduced in ICG-IBA-RGD treated mice after laser irradiation as compared to those in the other three groups (Fig. 6). These results confirm that ICG-IBA-RGD has good performance in tumor PTT.

## Conclusions

In summary, we successfully designed and constructed a superior albumin-binding photothermal agent ICG-IBA-RGD for targeted tumor imaging and photothermal therapy. ICG-IBA-RGD shows enhanced photothermal conversion efficiency and low toxicity both at the cellular and animal levels. The increased and long-lasting accumulation in tumor of ICG-IBA-RGD was observed in the imaging experiment, which will benefit for targeted photothermal therapy. Actually, the phototherapy study indeed demonstrated that ICG-IBA-RGD could efficiently eliminate tumor without recurrence within 14 d. Collectively, we report here a superior albumin-binding photothermal agent ICG-IBA-RGD and bring a new insight into the development of excellent phototheranostics for practical cancer theranostics.

## Ethical statement

All animal procedures were performed in accordance with the Guidelines for Care and Use of Laboratory Animals of Central South University and approved by the Animal Ethics Committee, The Second Xiangya Hospital, Central South University, China.

## Conflicts of interest

There are no conflicts to declare.

## Supplementary Material

RA-011-D0RA09653A-s001
