# Supplementary material for: Novel albumin-binding photothermal agent ICG-IBA-RGD for targeted fluorescent imaging and photothermal therapy of cancer
Source: RSC Adv. 2021 Feb 11;11(13):7226–30. doi: 10.1039/d0ra09653a (PMC8695055; doi:10.1039/d0ra09653a)
Supplement: RA-011-D0RA09653A-s001 [file RA-011-D0RA09653A-s001.pdf]

Electronic Supplementary Material (ESI) for RSC Advances.
This journal is © The Royal Society of Chemistry 2021

Supplementary data for

Novel Albumin-binding Photothermal Agent ICG-IBA-RGD for Targeted Fluorescent Imaging and Photothermal Therapy of Cancer

Cheng Yu[a], Enhua Xiao[a], Pengfei Xu[c], Jingjing Lin[b], Linan Hu[a], Jie Zhang[a], Shengzhen Dai[a], Zhuyuan Ding[a], Yuyang Xiao[a] and Zhu Chen[a]*

[a]Departments of Radiology, The Second Xiangya Hospital, Central South University, Changsha, Hunan 410011, P.R. China;

[b]Department of Psychiatry, The Second Xiangya Hospital, Central South University; National Clinical Research Center for Mental Disorders; Hunan Medical Center for Mental Health, Changsha, Hunan 410011, P.R. China;

[c]Institute of Clinical Pharmacy & Pharmacology, Jining First People's Hospital, Jining Medical University, Jining 272000, P.R. China;

ORCID number: Cheng Yu (0000-0001-6341-5834); Zhu Chen* (0000-0002-5639-8949).

Experimental

Materials

All commercial chemicals were purchased from commercial suppliers and used without further purification. All solvents were purified before use. Breast cancercell line 4T1 was purchased from the Type Culture Collection of the Chinese Academy of Sciences, Shanghai, China. Dulbecco's modified Eagle's medium (DMEM) were purchased from Sigma-Aldrich. Other medium

components were obtained from Sigma-Aldrich too. Fetal bovine serum (FBS) was obtained from Hyclone Laboratories Inc. Hydrogen peroxide 30% was bought from Sinopharm Chemical Reagent Co., Ltd (Shanghai, China). Fluorescence imaging of 4T1 cells were obtained using Olympus FV1000 confocal fluorescence microscope. Electrospray ionization mass spectra (ESI-MS) were collected on Agilent 6460 Triple Quadrupole LC/MS instrument.

Synthesis of ICG-L1

ICG-NHS (8 mg, 1 eq) in 3mL anhydrous N, N-dimethylformamide (DMF) was stirred with compound 1 (5 mg， 1.1 eq) with the addition of DIPEA (3 eq). Then, the mixture was stirred under an N^2 atmosphere at room temperature. The reaction was monitored by TLC analysis and it was completed in 4 hours. The reaction mixture was concentrated in vacuo and the residue was purified by Flash chromatography to give ICG-L1 (8.5 mg, 73% yield)

Synthesis of ICG-IBA

Deprotection of Boc of ICG-L1 (6 mg, 1 eq) was done at RT using neat trifluoracetic acid (TFA) in a total volume of 2 mL. Completion of deprotection was monitored by HPLC. TFA was removed under argon flow and 2 mL anhydrous N, N-dimethylformamide (DMF) was added. Then, 4-(p-iodophenyl) butyric acid (1.1 eq), (1-[bis(dimethylamino)methylene]-1H-1,2,3-triazolo[4,5-b]pyridinium 3-oxide hexafluorophosphate) (HATU, 1.2 eq), and diisopropylethylamine (DIPEA, 5 eq) were added under Argon flow. Then, the reaction mixture was monitored by analytical HPLC and purified by Pro-HPLC. LC-MS analysis confirmed mass of 1350 [M]+ with an isolated yield of 53% (3.5 mg)

Synthesis of ICG-IBA-RGD

ICG-IBA (1 eq, 3.5 mg) was dissolved in 0.5 mL of DMSO. RGD-SH (1.1 eq) was dissolved in 0.5 mL of de-gassed 0.1% sodium ascorbate (w/v) in phosphate buffer-saline (PBS) and added to the

reaction solution. The reaction was stirred at RT for 2 h. The reaction mixture was monitored by analytical HPLC and purified by Pro-HPLC. LC-MS analysis confirmed mass of 1929.95 [M]+ with an isolated yield of 62% (3 mg).

Photothermal Property of ICG-IBA-RGD

ICG-IBA-RGDs dispersed in deionized water at different concentrations (0、12.5、25、50、100、200 μg/mL) were exposed to 808 nm laser irradiation with a power density of 1.0 W/cm^2. The temperature at different time periods was measured and recorded by TM500 digital thermometer.

4T1 Cell Culture and *In Vitro* Cytotoxicity Assay

4T1 murine breast cancer cells were cultured in RPMI-1640 medium containing 10% fetal bovine serum and 1% penicillin/streptomycin at 37 ℃ under 5% CO2. The cytotoxicity of **ICG-IBA-RGD** was evaluated by Cell Counting Kit-8 (CCK-8, Dojindo, Japan) assay. 4T1 cells were seeded into 96-well plate at a density of 5000 cells per well and cultured at 37 ℃ overnight. Subsequently, 100 μL dispersion of **ICG-IBA-RGD**s was added to each well with different concentrations (0, 12.5, 25, 50, 100, 200, and 400 μg/mL) and then incubated for 24 h. Then 10 μL of CCK-8 was added to each well and further incubated at 37 ℃ for another 2 h. Finally, the 96-well plates were placed in a microplate reader (TECAN, Switzerland) to measure the absorbance at 450nm.

***In Vitro* PTT**

The photothermal effect *in vitro* was evaluated by live/dead cell staining analysis. 4T1 cells were seeded in a 24-well plate at a density of 5 x 105 cells per well and incubated with different concentrations of **ICG-IBA-RGD** suspensions (0、12.5、25、50、100、200 μg/mL). After irradiated with NIR laser (808nm, 1.0W/cm^2) for 5 min, the fluorescein diacetate (FDA) and propidium iodide (PI) were used to stain live and dead 4T1 cells for visualization, respectively.

Fluorescence images of the stained cells were obtained using an inverted fluorescence microscope (Leica DMIL LED+EC3).

Tumor Model

Female Balb/c nude mice (6 weeks old, Department of Experimental Animals of Central South University) were used to establish 4T1 xenograft tumor models. 4T1 cells (2 × 106) suspended in 100 µL of PBS were subcutaneously injected to the right armpit of each mouse. The mice were used for *in vivo* experiments when the tumor volume reached approximately 75 mm^3. The tumor volume was defined as $V=LW^2/2$, where the L and W are the longest and shortest diameters respectively.

***In Vivo* Imaging and Biodistribution**

When the tumor volumes reached 75 mm^3, the tumor-bearing nude mice were divided into two groups (n = 3) at random. Subsequently, **ICG-RGD** and **ICG-IBA-RGD** solution (equal dye concentration) were intravenously injected into the tails of mice. Fluorescent pictures at different time points were captured using a Kodak *In Vivo* FX Pro Imaging System. At 48 h post-administration, the mice were immediately euthanized. The tumors and major organs of mice were excised for testing their fluorescence intensities.

***In Vivo* PTT**

The 4T1 tumor-bearing nude mice were used for *in vivo* PTT when the tumor volume reached about 75 mm^3. The mice were divided randomly into 4 groups (n=4 in each group): (1) PBS only (2) Laser only (3) **ICG-IBA-RGD** (1mg/ml) (4) **ICG-IBA-RGD** (1mg/ml) + laser. Groups 1, 3, and 4 were intravenously injected with 100 µL of solution. NIR fluorescence imaging was first performed prior to PTT for outlining the tumor and further determine the range of irradiation field. After 24 h post-injection, the tumor sites of mice in groups 2 and 4 were irradiated with 808 nm laser (1.0W/cm^2,

10 min). One day after laser irradiation, one mouse from each group was randomly selected and sacrificed, and the tumor tissues, main organs and eyeball blood were collected. Eyeball blood is used to detect the liver and kidney function. The main organ tissues were stained with Hematoxylin-Eosin (H&E), and the tumor tissues were stained with H&E, TdT-mediated dUTP Nick-End Labeling (TUNEL) and Proliferating Cell Nuclear Antigen (PCNA). Animal weight and tumor volume were recorded every 2 d.

Statistics

The data were presented as mean values ± SD. Student t-test was utilized to calculate the statistical significance. Any p-value <0.05 were considered to be statistically significant.

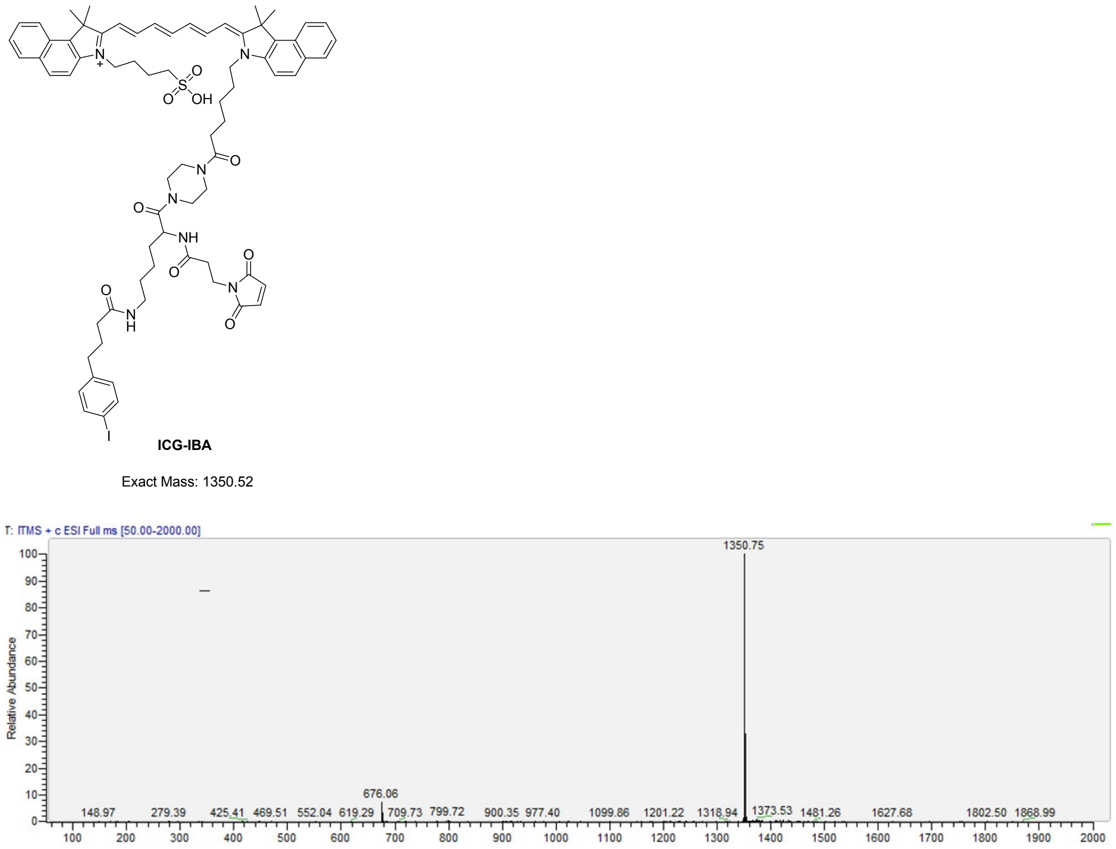

Figure S1. MS spectroscopy of **ICG-IBA**.

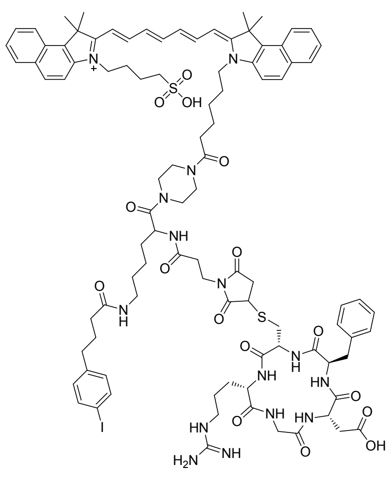

ICG-IBA-RGD

Exact Mass: 1928.74

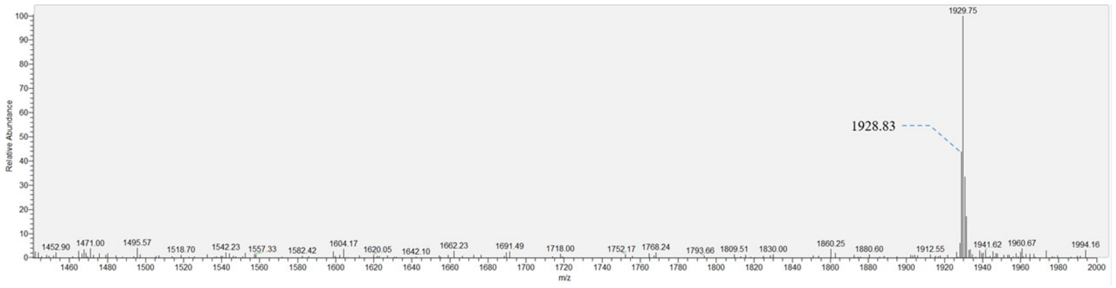

Figure S2. MS spectroscopy of **ICG-IBA**-**RGD**.

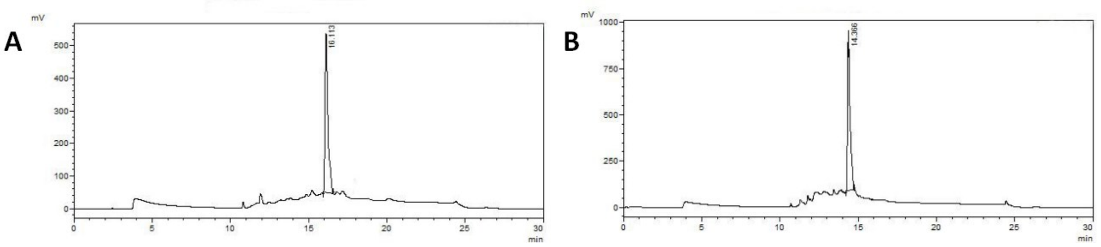

Figure S3. (A) HPLC chromatogram (254 nm) of **ICG-IBA**. (B) HPLC chromatogram (254 nm) of the reaction of ICG-IBA with RGD.

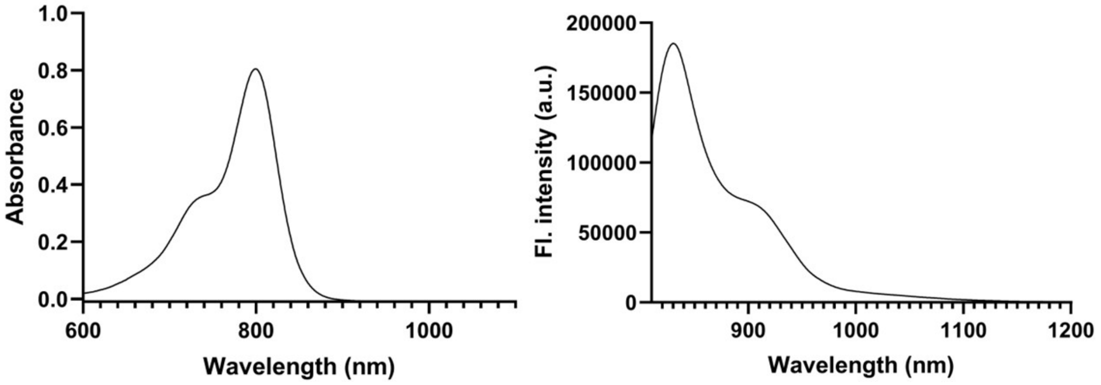

Figure S4. The UV/Vis absorption spectra and fluorescence spectra of **ICG-IBA-RGD** (5 μM) in 5% HSA.

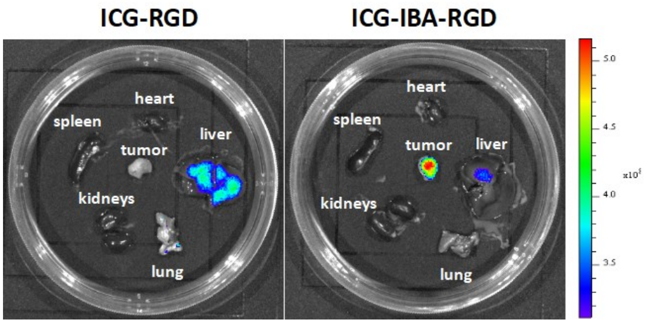

Figure S5. NIR fluorescence images of major organs and tumors after 48 h post-injection of **ICG-RGD** and **ICG-IBA-RGD**.

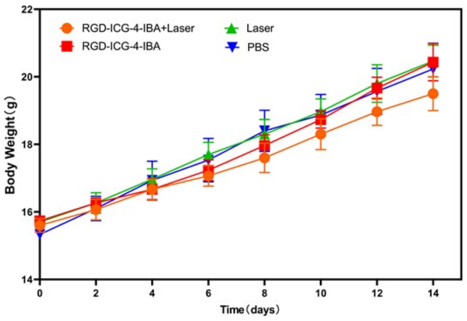

Figure S6. The body weight of mice after various treatments.

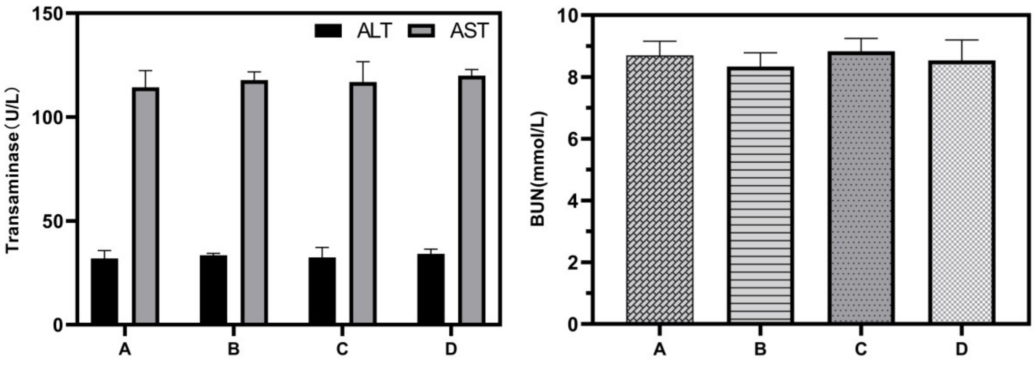

Figure S7. Liver and kidney function markers of mice after being treated with (A) PBS, (B) Laser, (C) **ICG-IBA-RGD**, and (D) **ICG-IBA-RGD** + laser, respectively.

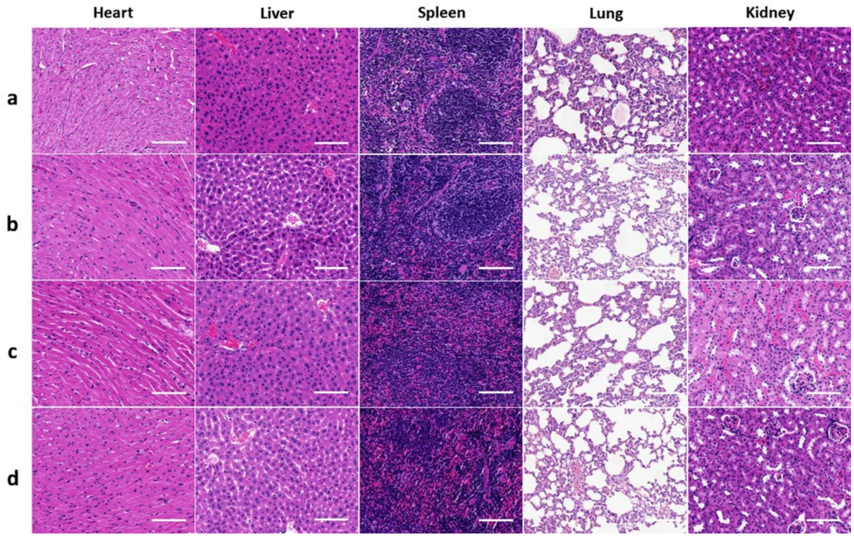

Figure S8. H&E staining images of the major organs including heart, liver, spleen, lung and kidney after the mice were sacrificed on the second day after injection. (a, PBS; b, Laser; c, ICG-IBA-RGD; d, ICG-IBA-RGD + laser)
